# Object Surface Recognition Based on Standing Waves in Acoustic Signals

**DOI:** 10.3389/frobt.2022.872964

**Published:** 2022-04-25

**Authors:** Makoto Kumon, Rikuto Fukunaga, Tomoya Manabe, Kei Nakatsuma

**Affiliations:** ^1^ Faculty of Advanced Science and Technology, Kumamoto University, Kumamoto, Japan; ^2^ Graduate School of Science and Technology, Kumamoto University, Kumamoto, Japan; ^3^ Faculty of Engineering, Kumamoto University, Kumamoto, Japan

**Keywords:** robot audition, object recognition, echo location, standing wave, distance spectrum

## Abstract

This paper proposes the use of the standing waves created by the interference between transmitted and reflected acoustic signals to recognize the size and the shape of a target object. This study shows that the profile of the distance spectrum generated by the interference encodes not only the distance to the target, but also the distance to the edges of the target surface. To recognize the extent of the surface, a high-resolution distance spectrum is proposed, and a method to estimate the points on the edges by incorporating observations from multiple measurement is introduced. Numerical simulations validated the approach and showed that the method worked even in the presence of noise. Experimental results are also shown to verify that the method works in a real environment.

## 1 Introduction

Robots are expected to be used in various environments, which requires them to have situational awareness and the autonomy to make proper decisions accordingly. Thus, environment recognition is one of the key functions for autonomous robots.

Visual sensors such as cameras and light detection and ranging devices have been intensively investigated for this purpose. Especially for three-dimensional recognition with visual sensors, cameras with a depth or range sensing function are developed; for example, a time-of-flight camera was incorporated for mapping the environment May et al. (2009), a structured-light approach was proposed for the range recognition Boyer and Kak (1987), and a light-section technique Wang and Wong (2003) was introduced for accurate range measurement of targets nearby. Another candidate is acoustic sensors *e.g.*, ultrasonic range sensors for obstacle detection, and sound navigation and ranging for underwater guidance. While the measurement resolution of acoustic sensors is not as accurate as that of visual sensors, acoustic measurements can compensate visual methods because acoustic observation is robust to visual interference, such as changing lighting conditions, challenging medium quality due to small particles such as fog or dust, and occlusions. Specular surfaces or transparent objects (*e.g.* glass objects) cause “blind spots” in visual measurements, and acoustic methods can be employed to detect such objects.

Popular acoustic sensors use ultrasonic signals. Ultrasonic sensors are “active” in the sense that they emit the signal and receive a reflected signal. The features of the reflected signal, such as time-of-flight Santamaria and Arkin (1995); Kuc (2008), the spectrum Reijniers and Peremans (2007), and the envelope Egaña et al. (2008), are analyzed to extract information about the object. Such echolocation architecture can be also integrated into a robotic localization system (Steckel et al. (2012); Lim and Leonard (2000).

The detectable range of high-frequency signals with a short wavelength is limited because of attenuation during propagation; the common detection range of commercial ultrasonic sensors is up to several meters. In contrast, audible signals with a frequency of 20–20 kHz have longer wavelengths, and a longer penetration distance than ultrasonic signals, which may improve the detection range. An example of the use of such audible signals is visually impaired people using “clicking” tones to recognize their environments and objects Kolarik et al. (2014). Users trained with clicking have a detection range of more than 30 m Pelegrin-Garcia et al. (2018), and they can recognize not only the distance to targets but also their shapes Thaler et al. (2018).

Uebo et al. (2009) proposed to the use of audible signals to estimate the distance based on standing waves produced by interference between the transmitted and reflected signals; the power spectrum of the standing wave has a periodic structure in the frequency domain, and its period encodes the distance between the sensor and the object. Kishinami et al. (2020) proposed a method for recognizing multiple objects by using a distance estimation based on a standing wave incorporating the phase information with reliable frequency selection Takao et al. (2017).

Shape and orientation of the target object are useful features to recognize. For example, a method was proposed for recognizing the features of the target, such as flat or sharp corner from the profile of the reflected ultrasonic pulses Smith, (2001). Another approach is to estimate the shape of objects given a model of its shape (Santamaria and Arkin (1995)). Recently, BatVision (Christensen et al. (2020)) and CatChatter (Tracy and Kottege (2021)) proposed incorporation of statistical learning techniques to reconstruct a depth image from acoustic echoes to recognize complicated shapes.

Because a wide-beam audible signal can cover a large area with long penetration, this paper proposes extending the distance recognition based on the standing wave to recognize the shape and the orientation of the target of interest. Authors (Kumon et al. (2021)) previously reported that the profile of a distant spectrum (Uebo et al. (2009)) encodes not only the distance to the target but also the distance to the edges of the target surface; peaks in the distance spectrum profile correspond to reflections from points on the edges. Because the distance to the edge may slightly differ to that to the surface, it is important for the distance spectrum to have sufficient resolution to distinguish them. To recognize those distances, this paper proposes the use of a high-resolution distance spectrum to detect such peaks clearly. With this high-resolution distance spectrum, the surface recognition framework in the previous work (Kumon et al. (2021)) can improve the estimate. This paper also proposes the use of multiple observations at different locations to reconstruct the shape.

The rest of this paper is organized as follows. Distance measurements based on the standing wave in an acoustic signal, as proposed by Uebo (Uebo et al. (2009)), are briefly summarized in Section 2. The distance spectrum profile is examined in Section 3, with reference to our previous work (Kumon et al. (2021)), and a method to estimate the shape of the target object is proposed. Then, we describe how the proposed was validated by numerical simulations and experiments (Section 4). Conclusions are given in Section 5.

## 2 Using a Standing Wave for Distance Measurement

This section describes the method for estimating the distance to an object using acoustic signals as proposed by Uebo (Uebo et al. (2009)).

**FIGURE 1:**
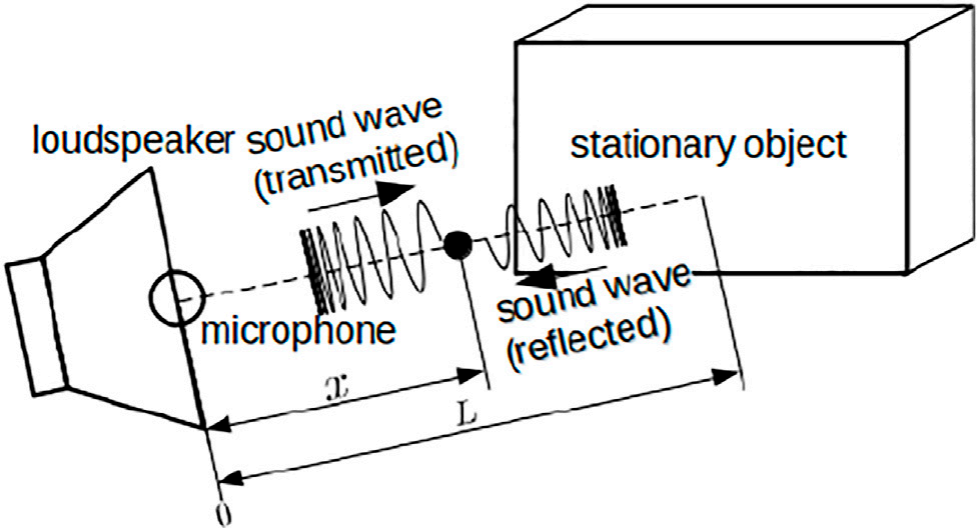
Schematic of model for distance estimation based on standing wave.

Figure 1 shows a schematic of the distance estimation method based on a standing wave. The signal transmitted from a speaker at time *t* to a point *x* is denoted as *v*_Tr_ (*t*, *x*). Further, we assume that the signal is a linear chirp given by

$$v_{Tr}\left(t,x\right)=Ae^{j\left(2\pi \int_{0}^{t-\|x\|/c}f\left(\tau \right)d\tau +\theta \right)},$$ (1)

where *f*(*τ*) is the instantaneous frequency defined as

$$f\left(\tau \right)=\frac{f_{w}}{T}\tau +f_{1},$$ (2)

and *A*, *c*, and *θ* represent the amplitude, speed of sound and phase, respectively; and *T*, *f*_1_, *f*_*N*_, and *f*_*w*_ represent the duration, lowest frequency, highest frequency, and bandwidth *f*_*w*_ = *f*_*N*_ − *f*_1_, respectively.

The signal reflected from a stationary object that locates at the distance *L* from the microphone is denoted as *v*_Ref_(*t*, *x*), and its assumed model is as follows:

$$v_{\text{Ref}}\left(t,x\right)=A\gamma e^{j\phi }e^{j\left(2\pi \int_{0}^{t-\left(2L-\|x\|\right)/c}f\left(\tau \right)d\tau +\theta \right)},$$ (3)

where *γ* and *ϕ* are reflection parameters that are constant over the frequency range of interest. The mixed signal *v*_C_ (*t*, 0) at the origin (*x* = 0), where a microphone is located, can be computed as *v*_C_ (*t*, 0) = *v*_Tr_ (*t*, 0) + *v*_R_ (*t*, 0). The power of *v*_C_ (*t*, 0) becomes

$$|v_{C}\left(t,0\right){|}^{2}=|A{|}^{2}\left\{1+\gamma^{2}+2\gamma \cos\left(2\pi \frac{f_{W}}{T}\frac{2L}{c}t-2\pi \frac{f_{W}}{2T}{\left(\frac{2L}{c}\right)}^{2}+2\pi f_{1}\frac{2L}{c}-\phi \right)\right\}.$$ (4)

The first and the second terms of the right-hand side show the power of the transmitted and received signals, and they are constant. The cosine term shows the effect of the interference and it is independent of the initial phase *θ*. Substituting (2) into (4) gives, the power of *v*_C_ in the frequency domain as

$$p\left(f,0\right)=|A{|}^{2}\left\{1+\gamma^{2}+2\gamma \cos\left(\frac{4\pi L}{c}f+C_{0}\right)\right\},$$ (5)

where *C*_0_ is a constant term for the interference. As (5) is a biased cosine function, the power spectrum of an unbiased *p* (*f*, 0) over the frequency range (*f*_1_, *f*_*N*_) forms an impulsive peak at the frequency of the cosine function. By rescaling this frequency with a factor $\frac{c}{2}$ , the power spectrum becomes the “distance spectrum” because it has a peak at *L* that represents the distance to the object.

## 3 Object Recognition Using Distance Spectrum Profile

### 3.1 Disk Model

**FIGURE 2:**
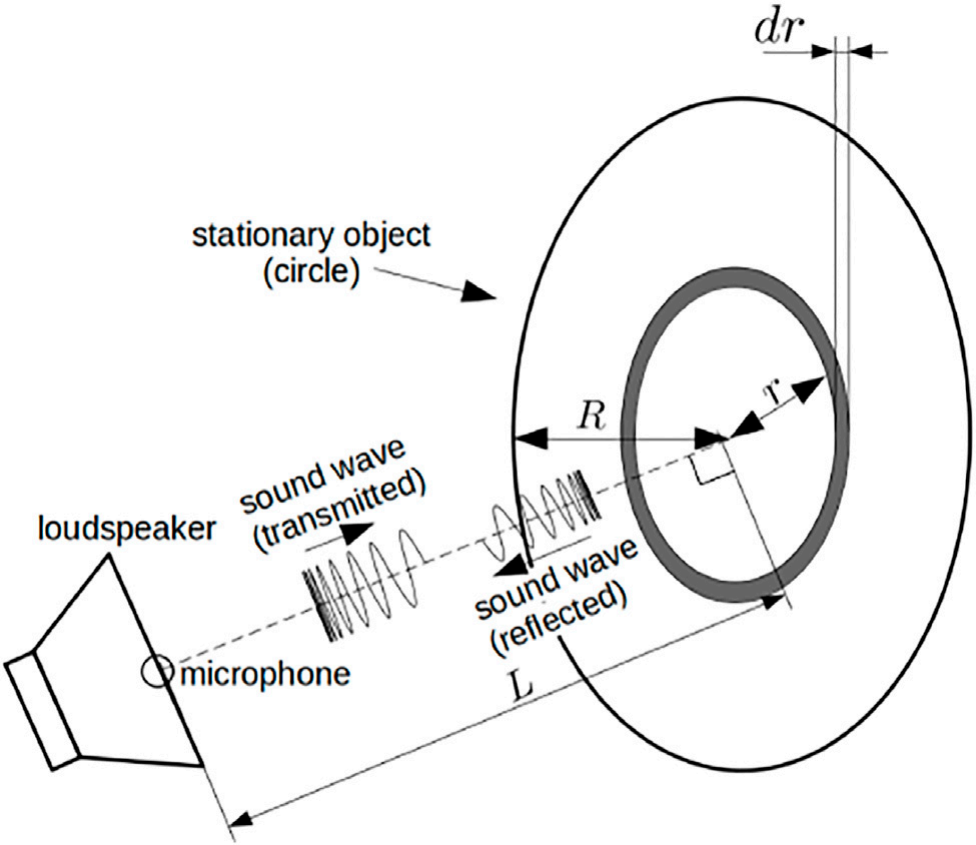
Schematic of standing wave model with disk.

The distance spectrum described in the previous section is based on a single reflected signal, which models the target object as a point. However, common objects in the real environment are difficult to approximate as points, and reflected signals from such objects can be affected by their spatial structure. To take the spatial effect into account, a disk reflection model is introduced (Figure 2) as previously reported (Kumon, M. et al. (2021)).

Consider a disk with a radius *R* located at a distance *L* as the target object, as shown in Figure 2. For simplicity, the observation point is located on the line that perpendicularly passes through the center of the disk. Note that the chirp signal during a short period can be approximated as a sine wave and that the phase of the transmitted signal does not affect the distance spectrum. For simplicity, the transmitted signal is modeled as a unit pure tone sin *ωt* in the following analysis. The reflected acoustic signal with an angular frequency *ω* from a ring that has a radius *r* and width d*r* at the origin *x* = 0 is denoted as *v*_Ref,*r*,*ω*_(*t*, 0)d*r*. Under the assumption that the gain coefficient and the phase shift by the reflection are constant over the whole frequency range as in (Uebo, T. et al. (2009)), we modeled the reflected signal by applying Lambert’s law (Kuttruff (1995)) and inverse distance law as follows;

$$\begin{array}{ll} v_{Ref,r,\omega }\left(t,0\right)dr & =\gamma \sin\left(\omega \left(t-\frac{\sqrt{L^{2}+r^{2}}}{c}\right)+\phi \right)\times \underset{{Lambert}^{\prime }s\;law}{\frac{L}{\sqrt{L^{2}+r^{2}}}}\\ & \;\times \underset{Inverse\;distance\;law}{\frac{1}{\sqrt{L^{2}+r^{2}}}}\times \underset{Area\;ratio}{\frac{2\pi r}{\pi R^{2}}dr}. \end{array}$$ (6)

By integrating *v*_Ref,*r*,*ω*_(*t*, 0)d*r* of (6) over the disk, the reflected signal *v*_Ref,*ω*_(*t*) is computed as follows:

$$\begin{array}{ll} v_{\text{Ref},\omega }\left(t,0\right) & =\int_{0}^{R}v_{\text{Ref},r,\omega }\left(t,0\right)dr\\ & =\frac{2\gamma L}{R^{2}}\left\{C_{1}\sin\left(\omega t-\phi \right)+C_{2}\cos\left(\omega t-\phi \right)\right\}, \end{array}$$ (7)

where

$$\begin{array}{ccc} C_{1} & = & \int_{0}^{R}\frac{r}{L^{2}+r^{2}}\cos\left(\frac{\omega }{c}\sqrt{L^{2}+r^{2}}\right)dr\\ & = & C_{i}\left(\frac{\omega }{c}\sqrt{L^{2}+R^{2}}\right)-C_{i}\left(\frac{\omega }{c}L\right), \end{array}$$

and

$$C_{2}=S_{i}\left(\frac{\omega }{c}\sqrt{L^{2}+R^{2}}\right)-S_{i}\left(\frac{\omega }{c}L\right).$$

The term C_i_(⋅) and S_i_(⋅) are the cosine integral and the sine integral, respectively (Weisstein, (2002)):

$$\begin{array}{ccc} C_{i}\left(x\right) & = & \int_{0}^{x}\frac{1-\cos t}{t}dt,\\ S_{i}\left(x\right) & = & \int_{0}^{x}\frac{\sin t}{t}dt. \end{array}$$

The power of the measured signal sin *ωt* + *v*_Ref,*ω*_(*t*, 0) at the origin can be written as

$$\begin{array}{ll} p\left(\omega ,0\right) & ={\left[1+\frac{2\gamma L}{R^{2}}\left\{C_{1}\cos\phi +C_{2}\sin\phi \right\}\right]}^{2}\\ & \;+{\left[\frac{2\gamma L}{R^{2}}\left\{-C_{1}\sin\phi +C_{2}\cos\phi \right\}\right]}^{2}\\ & =1+\alpha^{2}\left({C_{1}}^{2}+{C_{2}}^{2}\right)+2\alpha \left(C_{1}\cos\phi +C_{2}\sin\phi \right), \end{array}$$

where $\alpha =\frac{2\gamma L}{R^{2}}$ .

The cosine integral C_i_(*x*) and the sine integral S_i_(*x*) can be approximated for sufficiently large *x*, |*x*|≫ 1 (Airey (1937)) as

$$C_{i}\left(x\right)\approx \frac{\sin x}{x}-\frac{\cos x}{x^{2}},\;S_{i}\left(x\right)\approx \frac{\pi }{2}-\frac{\cos x}{x}-\frac{\sin x}{x^{2}}.$$ (8)

Let $a=\frac{\sqrt{L^{2}+R^{2}}}{c}$ and $b=\frac{L}{c}\;\left(a>b\right)$ . Using (8), the following approximation holds (see the Appendix for details):

$$\left\{\begin{array}{c} {C_{1}}^{2}+{C_{2}}^{2}\approx 0\;\\ C_{1}\cos\phi +C_{2}\sin\phi \approx \frac{\sin\left(a\omega -\phi \right)}{a\omega }-\frac{\sin\left(b\omega -\phi \right)}{b\omega }.\; \end{array}\right.$$ (9)

Finally, the power *p* (*ω*, 0) can be approximated as

$$p\left(\omega ,0\right)\approx 1+2\alpha \left(\frac{\sin\left(a\omega -\phi \right)}{a\omega }-\frac{\sin\left(b\omega -\phi \right)}{b\omega }\right).$$ (10)

The approximation (10) shows that the power spectrum *p* (*ω*, 0) can be approximated by a constant and two sinc functions with coefficients *a* and *b*. Recalling that *ω* is given over a bounded frequency range, the “power spectrum” of *p* (*ω*, 0) for *ω* forms the distance spectrum that has two peaks at *a* and *b* that correspond to the edge (*a*) and distance (*b*), respectively, of the disk.

**FIGURE 3:**
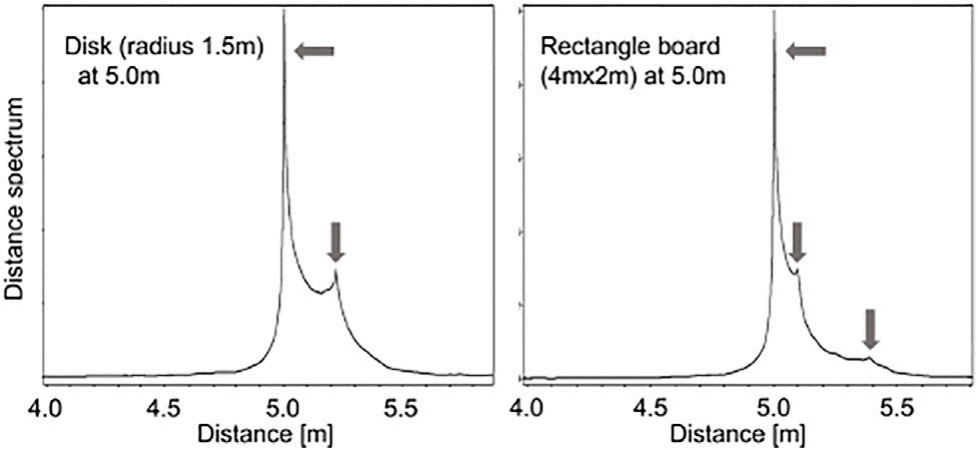
Example of a distance spectrum (left: 1.5 m radius disk at 5.0 m, right: 4.0 m×2.0 m rectangle at 5.0 m).

To verify this approximation model for the distance spectrum, a simple numerical acoustic simulation was conducted. In the simulation, a chirp signal was emitted from *L* = 5.0 m away from a disk of radius *R* = 1.5 m that had a reflection gain *γ* = 0.05 and a phase shift *ϕ* = *π* rad. The distance spectrum shown in the left figure of Figure 3 has two peaks at 5.0 and 5.22 m that correspond to the distance to the disk and the distance from its center to its edge, respectively $\left(\sqrt{5.0^{2}+1.5^{2}}\approx 5.22\right)$ . It is worth noting that this approach also works for a rectangle; a numerical result with a 4.0m×2.0 m rectangle board at 5.0 m shows three peaks corresponding to its distance and two edges (5.0 m, $\sqrt{5.0^{2}+{\left(\frac{2.0}{2}\right)}^{2}}\approx 5.10$ m and $\sqrt{5.0^{2}+{\left(\frac{4.0}{2}\right)}^{2}}\approx 5.39$ m).

### 3.2 High-Resolution Distance Spectrum

Because the distance spectrum can be computed using a Fourier transform of the power spectrum *p* (*f*, 0), a fast Fourier transform (FFT) (Cooley and Tukey, (1965)) is an effective method for this computation. The FFT-based conventional distance spectrum has a uniform resolution, and Uebo (Uebo et al. (2009)) showed that the highest resolution for the distance spectrum is given by $\frac{c}{2f_{W}}$ .

**FIGURE 4:**
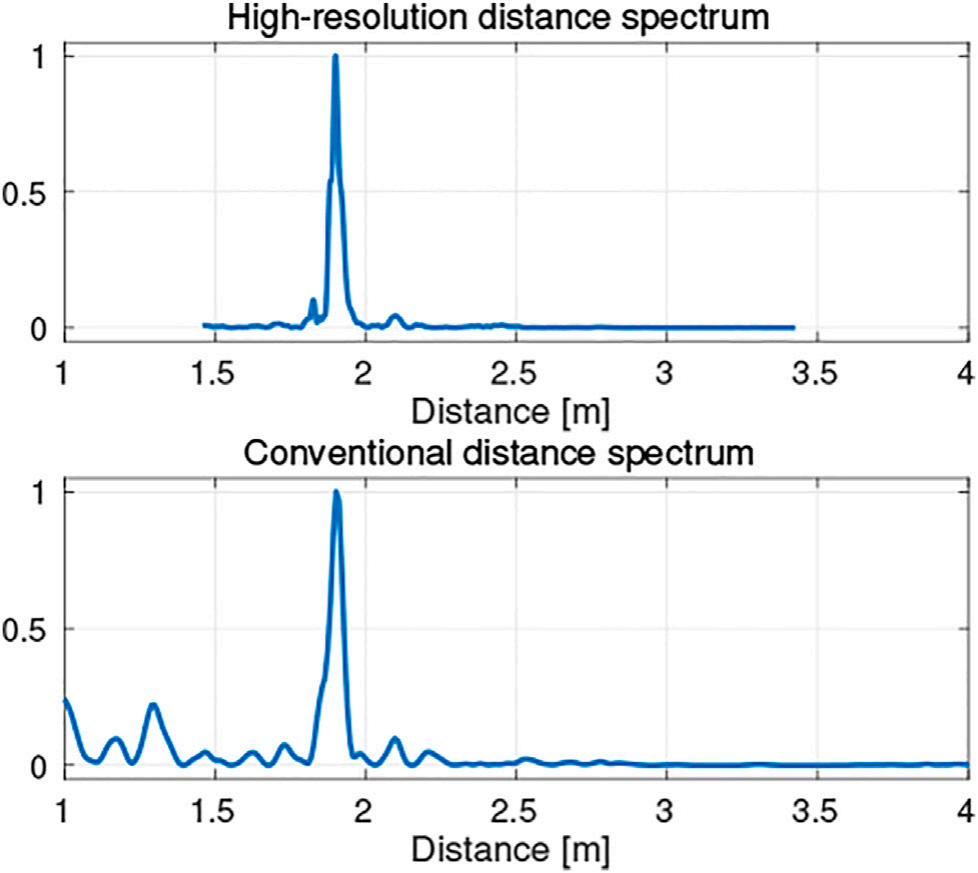
High-resolution distance spectrum.

As shown in Figure 3, the peaks corresponding to the edges of the target object are closely located around the most significant peak. This study developed a method to use a discrete Fourier transform (DFT) (Smith, (2002)) with dense query points around the first peak to compute the distance spectrum. Figure 4 shows examples of distance spectra, and the proposed result indicates distinct peaks while the conventional one contains false peaks. Note that the query points can be selected arbitrarily with the DFT, and that the obtained distance spectrum can have higher resolution than that obtained using a FFT. However, since the DFT approach requires more computation, in the present study the conventional FFT-based distance spectrum was cascaded to find a region of interest indicated by a major peak, and to then select the finer query points around the major peak so as to simultaneously obtain high resolution, and improve the computational efficiency.

### 3.3 Object Recognition Using Multiple Observations

A single observation gives a distance spectrum that provides the distance to the object and its edge(s). This subsection explains how multiple observations can be used to determine the shape and orientation of the object’s surface. Note that the proposed method is designed for the offline processing after all observations.

#### 3.3.1 Surface Detection

**FIGURE 5:**
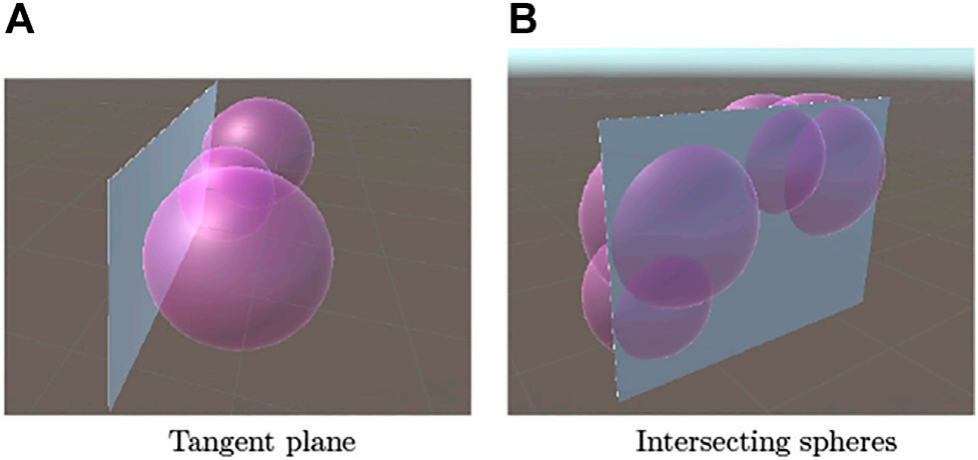
Spheres whose radii are estimated by distance spectra and the target planar object. **(A)** Tangent plane **(B)** Intersecting spheres.

Recall that the first peak in the distance spectrum contains information about the shortest distance to the target surface. Hence, the surface of the target object is on the common tangent plane of spheres whose radii are given by the most significant peaks in the distance spectra (Figure 5A).

This common tangent plane is computed as follows. The center of the sphere *i* and its radius are denoted as *O*_*i*_ and *r*_*i*_, respectively, and the unit normal vector for the plane is denoted as *n*. The distance between the plane and the origin is denoted by *d*. Then, the sphere and the plane can be modeled as

$$\|O_{i}-x\|=r_{i},$$ (11)

$$n^{T}x+d=0.$$ (12)

Because the contact point *x*_*i*_ satisfies both (11) and (12), ± *r*_*i*_ + *n* ^*T*^ *O*_*i*_ + *d* = 0 holds. For *N* observations, the equation can be combined in the following form:

O1T1O2T1⋯ONT1nd+±r1±r2⋮±rN=0,‖n‖=1. (13)

The relation (13) may not have a unique solution if the observed radius *r*_*i*_ is affected by measurement noise. To overcome this, the random sample consensus (RANSAC) (Fischler and Bollers, (1981)) approach was incorporated, in which randomly sampled subsets of *N* observations are used to form a sub-problem of (13), and the best-fit solution of the sub-problems is selected as the solution of (13). The sign of *r*_*i*_ is also determined in this process. The fitness to evaluate the solution for RANSAC is computed based on the residual of (13).

#### 3.3.2 Edge Recognition

**FIGURE 6:**
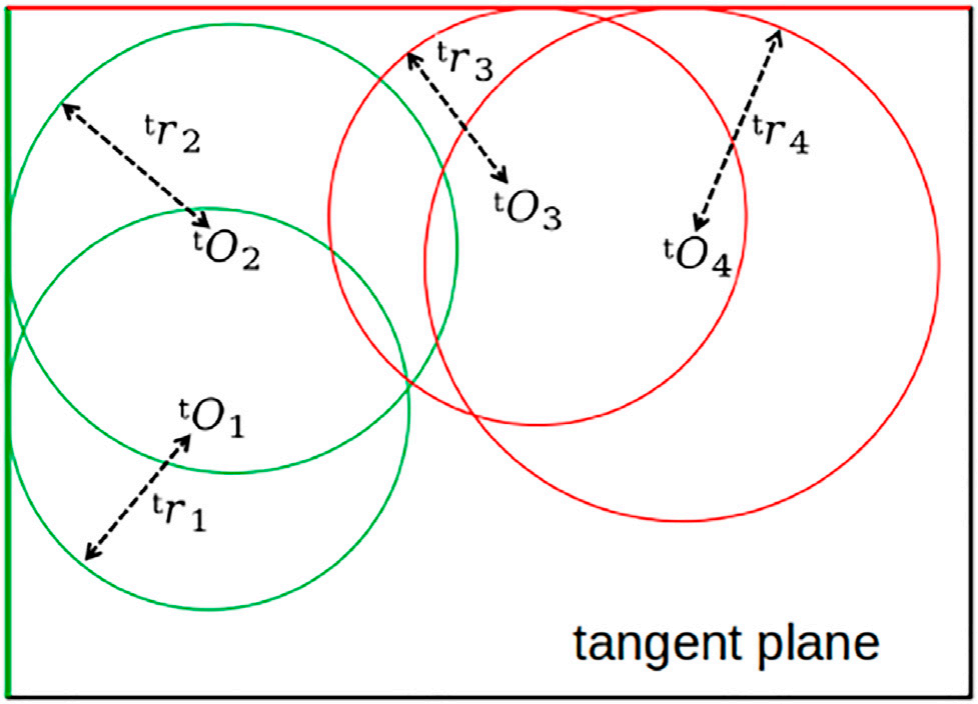
Example of spheres intersecting with tangent plane.

The second and subsequent peaks in the distance spectrum give the radii of spheres whose centers exist at the observation locations. These spheres intersect the common tangent plane obtained above as in Figure 5B. According to the disk model proposed in Section 3.1, these spheres contact the edge of the surface (Figure 6).

In this model, the shape of the surface is estimated by finding the contact points of spheres intersecting with the estimated tangent plane. This is an ill-posed problem because the contact points cannot be determined uniquely, but it may be acceptable to assume that multiple circles share the tangent line if that line corresponds to the actual edge of the target. Based on this idea, the developed method uses the frequency of the tangent lines contacting the circles as the likelihood of the surface’s edges. An algorithm inspired by Hough transform (Hough, (1962)) was used to solve this problem, as described below. The center of the circle *i* on the tangent plane is denoted as ^t^ *O*_*i*_ with a radius of ^t^ *r*_*i*_. The unit normal vector for the tangent line on the tangent plane is represented by ^t^ *n*(*ξ*) where *ξ* ∈ [0, 2*π*)rad indicates the angle between the line and the reference axis. The distance between the line and the origin is denoted as ^t^ *d* that hold, and then the contact point is dt=OiTttn+rit .

We introduce a function to evaluate the likelihood of *ξ* and *d* as

lξ,d=∑i=1Mexp−βd−dtξ;Oit,rit2, (14)

where *M* is the number of detected circles and *β* is a positive constant. Peaks of *l* (*ξ*, *d*) that are greater than a given threshold *l*_th_ are selected, and the corresponding parameters {(*ξ*, *d*)|*l* (*ξ*, *d*) ≥ *l*_th_, ∇*l* (*ξ*, *d*) = 0} are considered as the lines that form the shape of the target object on the tangent plane.

To determine the valid segments of the tangent lines, the contact points are computed. Let *ξ*_*j*_ and *d*_*j*_ be the estimated parameters for the line *j*. Then, the contact point between circle *i* and line *j*, denoted by ^t^ *p*_*i*,*j*_, is pi,jt=OiTttn⊥(ξj)n⊥t(ξj)+djnt(ξj) , where ^t^ *n* ^⊥^ is a vector perpendicular to ^t^ *n* on the tangent plane. Such contact points are considered as points on the edges of the target that allow us to recognize its shape.

## 4 Validation

### 4.1 Numerical Simulation

**FIGURE 7:**
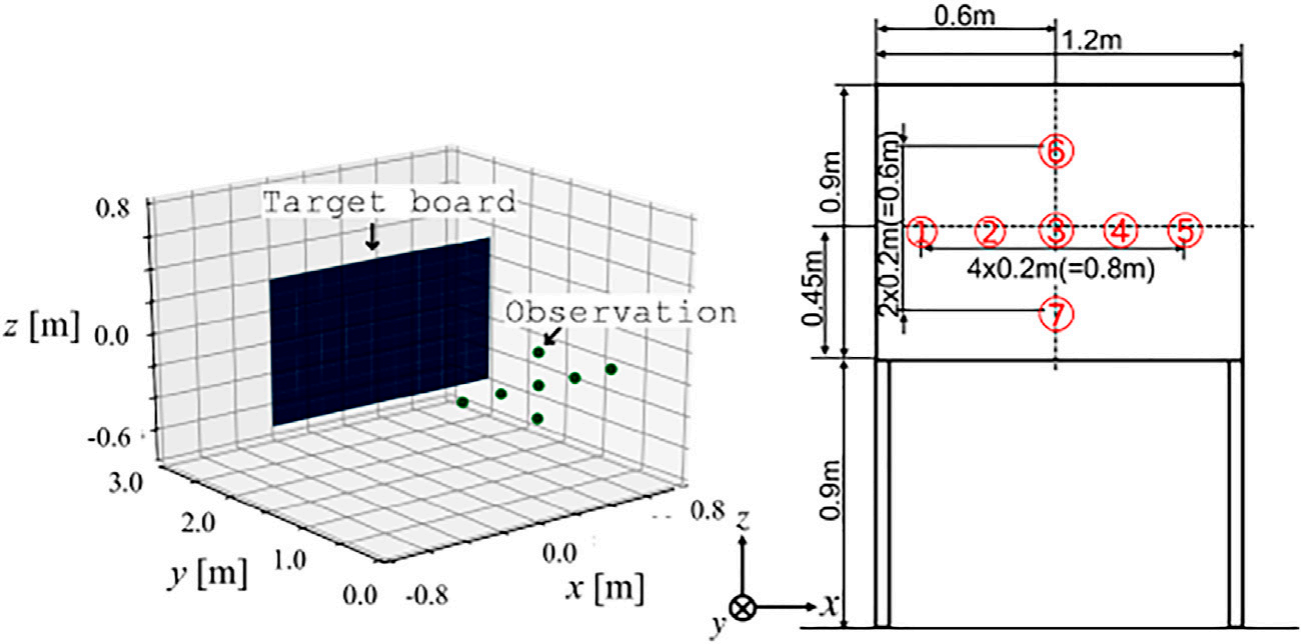
Target board and observation points for validation.

Acoustic numerical simulations were conducted to verify the developed method for recognizing the shape of a target. The considered target object was a board measuring 1.2 m × 0.9 m, as shown in Figure 7. A chirp signal in the 20 kHz frequency band was emitted from a transmitter, and the signal was sampled at 48 kHz. The emitter and the receiver were located at the same position, which was 2.0 m away from the board. Seven observations at the points shown in the figure were simulated. The surface located on the plane could be represented as (0, 1, 0)*x* − 2 = 0 in the coordinate frame of the figure. For efficient computation of the DFT to obtain a high-resolution distance spectrum, the non-equispaced FFT technique (Dutt and Rokhlin (1993)) was incorporated.

**FIGURE 8:**
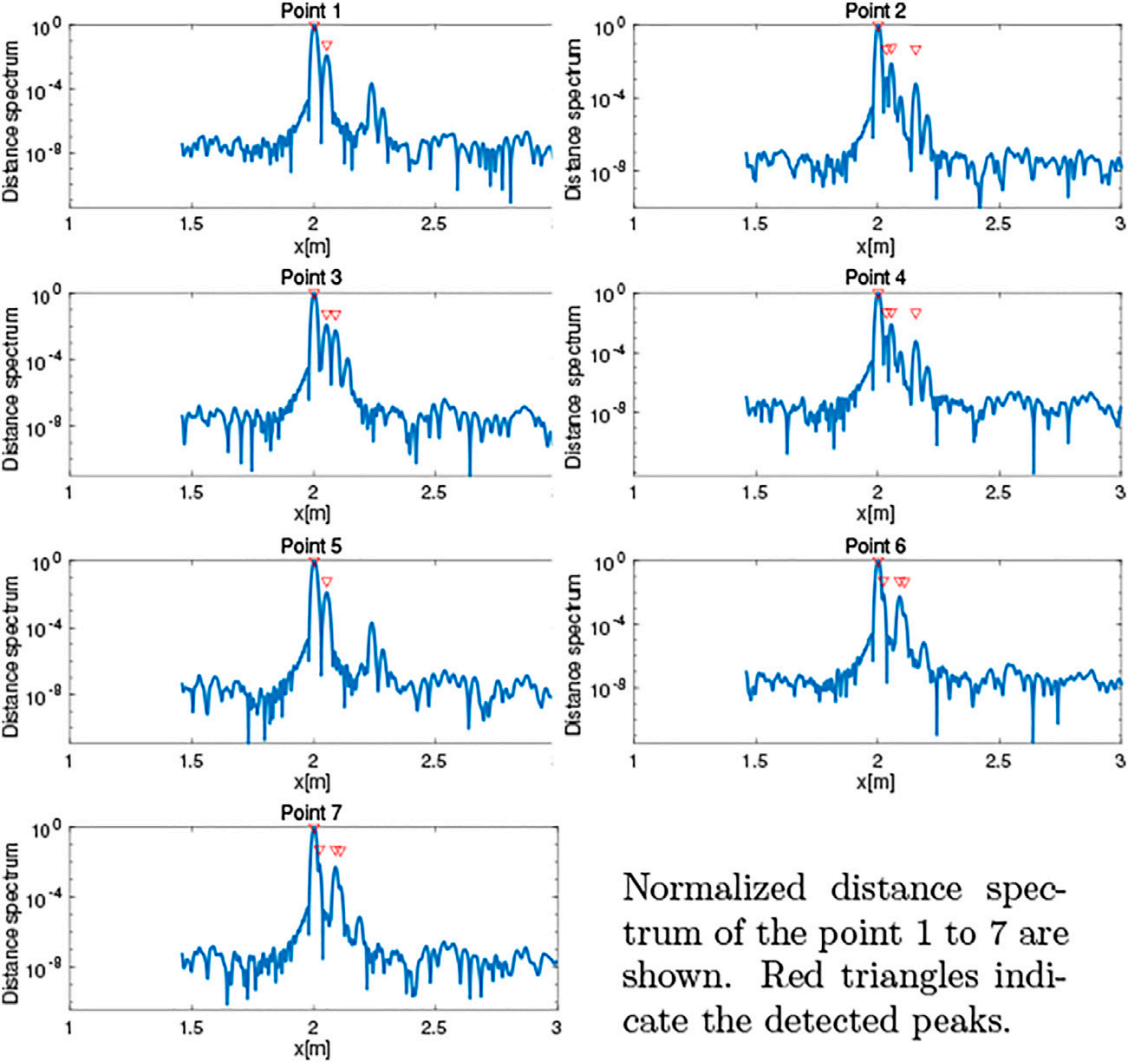
Distance spectrum obtained in simulation.

The obtained distance spectra are shown in Figure 8. The figure shows that the primary peaks that correspond to the distance to the board were extracted. Some of the peaks of the edges were also detected.

**FIGURE 9:**
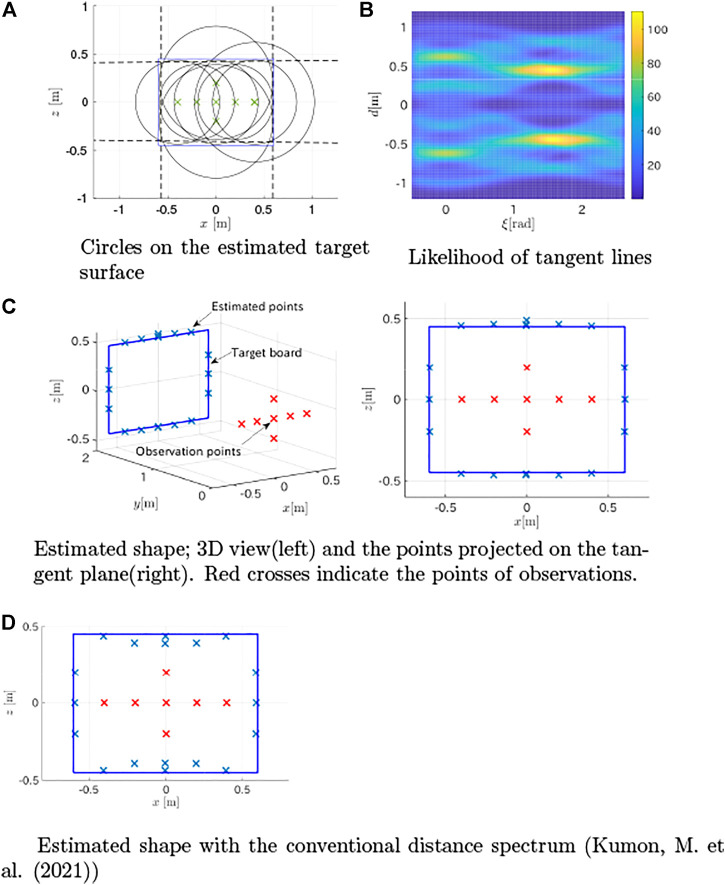
Target estimation results from simulation. **(A)** Circles on the estimated target surface **(B)** Likelihood of tangent lines **(C)** Estimated shape; 3D view(left) and the points projected on the tangent plane(right). Red crosses indicate the points of observations **(D)** Estimated shape with the conventional distance spectrum (Kumon, M. et. (2021)).

The method proposed in Section 3.1 was applied to estimate the plane containing the board from the first peaks in the distance spectra, and the estimated plane was (0.001, 0.9999, 0.000)*x* − 2.002 = 0. Then, the observations were fused using the method in Section 3.3 based on the estimated plane. The likelihood of tangent lines intersecting circles (Figure 9A) obtained by 14 is shown in Figure 9B. Then, the tangent lines with large likelihood values were estimated as the dotted lines shown in Figure 9A, and contact points were computed (crosses in Figure 9C). The figure shows that the method closely estimated the points of the edges. The distances between the 19 detected points and the nearest edges were computed as the estimation error. The mean absolute error was 0.0194 m with a standard deviation of 0.0297 m. This was better than the result (mean absolute error of 0.0417 m with a standard deviation of 0.0301 m, and shown in Figure 9D) obtained in our previous work (Kumon, M. et al. (2021)), which used the conventional FFT-based distance spectra.

**FIGURE 10:**
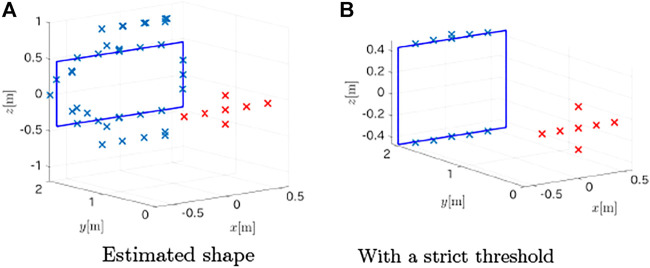
Estimated points of the target in the presence of noise (SNR = 60dB). **(A)** Estimated shape **(B)** With a strict threshold.

To evaluate the robustness of the method, it was tested under noisy measurement conditions. The signals were disturbed by uniform random noise whose magnitude was scaled to create a signal-to-noise ratio (SNR) of 60dB. The proposed method could accurately estimate the plane that contained the target as (0.001, 0.9999, 0.000)*x* − 2.002 = 0, but the estimated points for the target shape increased to 38, and some of the estimates were false positives, as shown in Figure 10A. The mean absolute error for these points was 0.1482 m with a standard deviation of 0.1441 m. By tuning the threshold to select the peaks from the distance spectra, the false detection rate could be reduced as shown in Figure 10B. The top and the bottom segments were estimated with a mean absolute error of 0.0290 m and a standard deviation of 0.037 m, but the method failed to detect the left and the right edges.

### 4.2 Experiment

**FIGURE 11:**
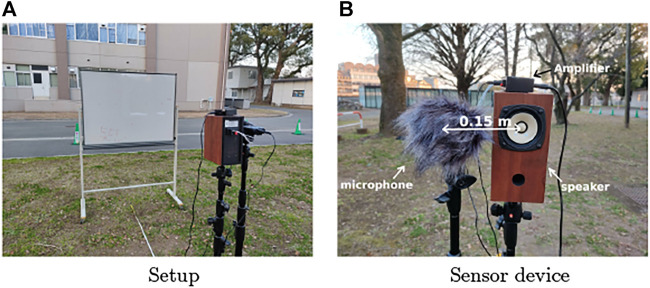
Photos of experimental setup and device. **(A)** Setup **(B)** Sensor device.

Next, we conducted experiments to show the validity of the proposed method. The target object was a board that had the same dimensions as in the numerical simulations depicted in Figure 11. A loudspeaker emitted a chirp signal, and the acoustic signal was recorded by a mobile audio recorder (Zoom, H4n Pro) that was located next to the speaker at a fixed distance of 0.15 m (Figure 11B). Although we placed the sensor device at multiple locations for the measurements instead of using an array of microphones, the principle still works for this case because the observation positions were precisely controlled. A synchronous averaging of 100 measurements was performed taking the background noise at the experiment site into account.

**FIGURE 12:**
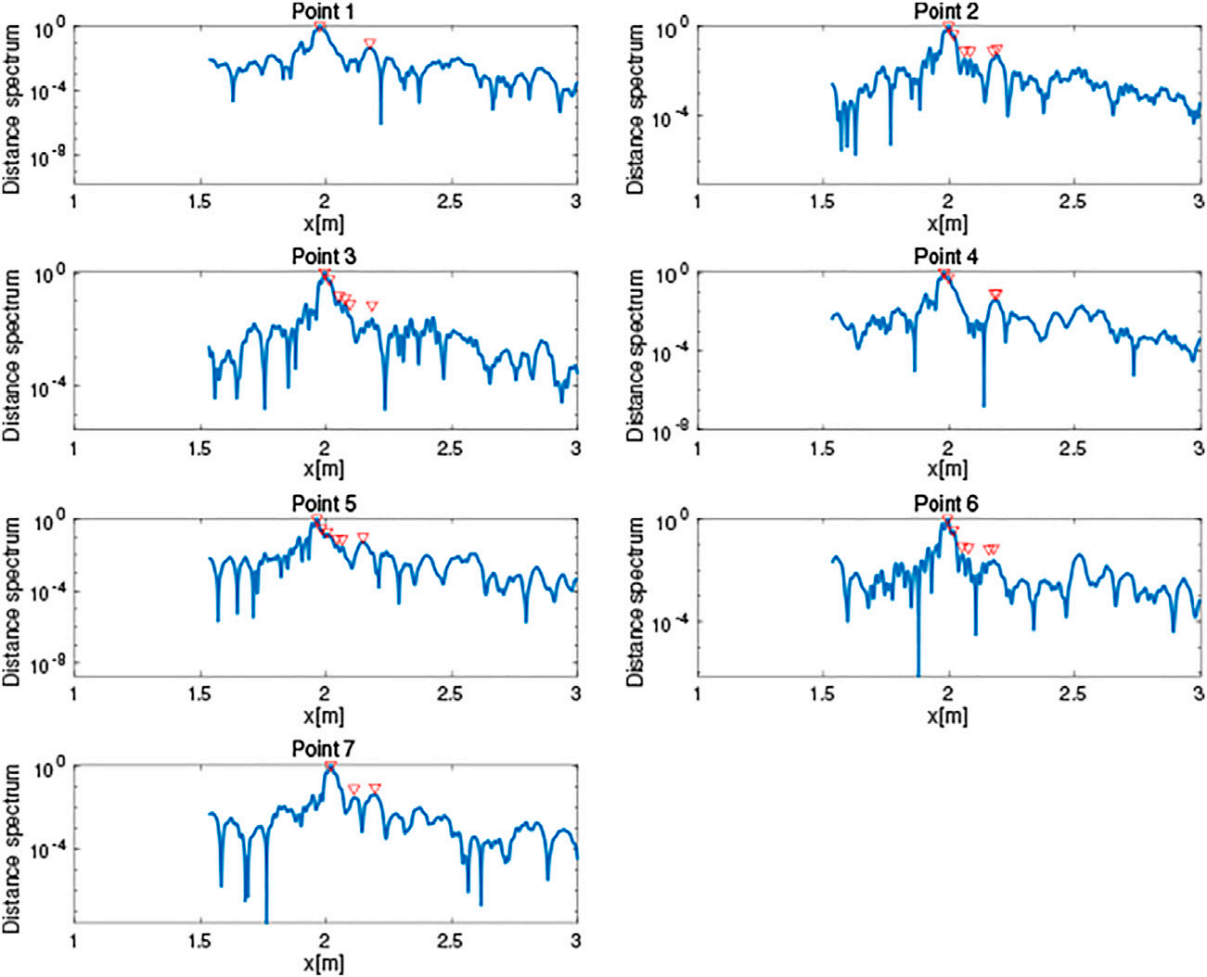
Distance spectrum obtained in experiment.

**FIGURE 13:**
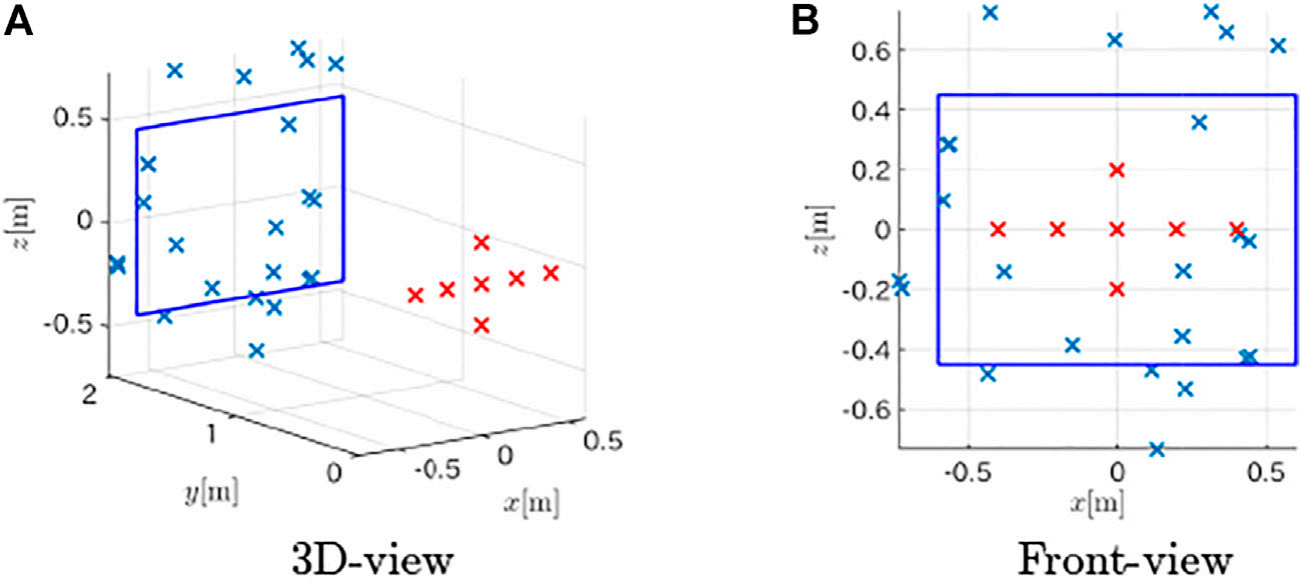
Estimated points on the target from the experiment. **(A)** 3D-view **(B)** Front-view.

The obtained distance spectra are shown in Figure 12, and the estimated plane was (−0.0399, 0.9973, 0.0619)*x* − 1.989 = 0; that is, the cosine similarity of the normal vector was 0.9973 and the *d* error was 0.011 m. As shown in Figure 13, 23 estimated edge points were detected and the mean deviation from the nearest edge and its standard deviation were 0.1410 and 0.0849 m, respectively. The surface plane was estimated accurately, and the shape of the board was roughly obtained, although the estimated points were scattered around the target. The limited performance for the shape estimation might be because of noise in the environment, installation errors of the device, and acoustic distortion by the microphone and speaker.

**FIGURE 14:**
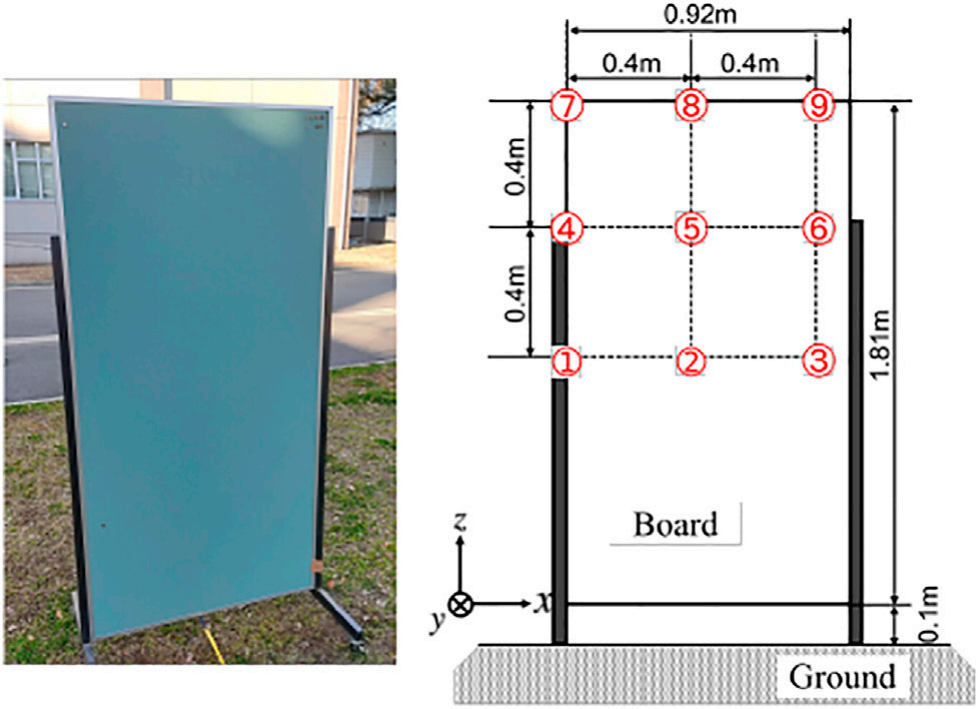
Experimental setup of a large board (left: a photo of the target, right: a diagram of the board dimension and the observation points).

Next, a larger board of 1.81 m × 0.92 m (Figure 14) was also used as another example to evaluate the proposed method. As shown in Figure 14, the bottom edge of the board was close to the ground which made the recognition challenging because the boundary of the target was difficult to detect. The observation pattern was designed to deviate from the center of the board to test in a more practical situation than in Figure 11.

**FIGURE 15:**
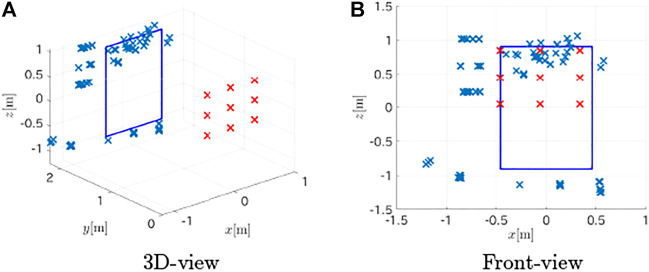
Estimated points on the large board target. **(A)** 3D-view **(B)** Front-view.

The same observation process as in the previous experiment was conducted to record acoustic observations, and the proposed method was used to estimate the target. The estimated plane containing the target board was (0.0521, 0.9948, 0.0871)*x* − 2.060 = 0; the cosine similarity of the normal vector was 0.9948 and the *d* error was 0.06 m; the performance to detect the target plane was at the same level as the previous experiment. Then, the method extracted 89 points as the points of the target as shown in Figure 15; 48 of those points corresponded to the top and the bottom edges while the rest 41 points located at the left of the board were false detections.

Because the method could detect the bottom segment that was distant from the center of the observation pattern, it can be concluded that the method has the potential to recognize the target with the observation pattern that partially covers the target. Of course, the large search space to detect distant boundaries may cause false detection as in the result, and further studies are necessary to determine the optimal choice of parameters.

## 5 Conclusion

This study shows that the profile of the distance spectrum encodes surface range information from the target, and a high-resolution distance spectrum can recognize the distance to the surface and that to the edges accurately. Cues from the high-resolution distance spectra can be used to estimate the shape of the target object surface. Numerical simulation validated the proposed approach, and the robustness of the method under noisy conditions was also verified. The method was also tested by experiments, which showed that the approach is feasible, although there remains room for performance improvement.

Future work is to account for outliers that occur under noisy measurement conditions, or for observations when the receivers are not pointing to the targets. Techniques to monitor the quality of the observations may be effective for such inappropriate readings. And the method needs to be tested with more targets in various environments. Another challenge is to extend the method for a more complicated environment, such as an indoor room that has significant reflections from several walls because it may become difficult to distinguish appropriate peaks of distance spectra. As multiple observations are used to estimate the target, a microphone array can be used to reduce the installation error among the microphones, to focus the reflections from the target of interest, and to reduce the noise.

## Data Availability

The data analyzed in this study is subject to the following licenses/restrictions: The data examined in this study was taken from the authors’ previous work; this is necessary to clarify the improvement by the proposed method. Requests to access these datasets should be directed to kumon@gpo.kumamoto-u.ac.jp.

## Appendix

The approximated cosine and sine integrals are denoted as $\bar{C_{i}}\left(x\right)$ and $\bar{S_{i}}\left(x\right)$ , that is,

$$\bar{C_{i}}\left(x\right)=\frac{\sin x}{x}-\frac{\cos x}{x^{2}},\;\bar{S_{i}}\left(x\right)=\frac{\pi }{2}-\frac{\cos x}{x}-\frac{\sin x}{x^{2}}.$$
(15)

Then, the following holds.

$$\begin{array}{ccc} & & {\bar{C_{i}}}^{2}\left(x\right)+{\bar{S_{i}}}^{2}\left(x\right)=\frac{\pi^{2}}{4}+\frac{1}{x^{2}}+\frac{1}{x^{4}}-\pi \left(\frac{\cos x}{x}+\frac{\sin x}{x^{2}}\right),\\ & & \bar{C_{i}}\left(x\right)\bar{C_{i}}\left(y\right)+\bar{S_{i}}\left(x\right)\bar{S_{i}}\left(y\right)=\frac{\pi^{2}}{4}-\frac{\pi }{2}\left(\frac{\cos y}{y}+\frac{\sin y}{y^{2}}+\frac{\cos x}{x}+\frac{\sin x}{x^{2}}\right). \end{array}$$

Furthermore,

$$\begin{array}{ccc} {C_{1}}^{2}+{C_{2}}^{2} & \approx & \frac{1}{{\left(a\omega \right)}^{2}}+\frac{1}{{\left(b\omega \right)}^{2}}+\frac{1}{{\left(a\omega \right)}^{4}}+\frac{1}{{\left(b\omega \right)}^{4}}-\frac{2}{ab\omega^{2}}\sqrt{1+\frac{a^{2}+b^{2}}{{\left(ab\omega \right)}^{2}}+\frac{1}{{\left(ab\right)}^{2}\omega^{4}}}\\ & & \;\times \sin\left(\left(b-a\right)\omega +\phi_{0}\right), \end{array}$$

where

$$\tan\phi_{0}=\frac{1+\frac{1}{ab\omega^{2}}}{\frac{1}{\omega }\left(\frac{1}{a}-\frac{1}{b}\right)}=\frac{ab\omega^{2}+1}{\left(b-a\right)\omega },$$

and

$$C_{1}\cos\phi +C_{2}\sin\phi \approx \frac{\sin\left(a\omega -\phi \right)}{a\omega }-\frac{\sin\left(b\omega -\phi \right)}{b\omega }-\frac{\cos\left(a\omega -\phi \right)}{{\left(a\omega \right)}^{2}}+\frac{\cos\left(b\omega -\phi \right)}{{\left(b\omega \right)}^{2}}.$$

For large *ω*, $\frac{1}{\omega^{2}}\ll 1$ and the above formulas give (9).
